# Core-Hole Excitation Spectra of the Oxides and Hydrates of Fullerene C_60_ and Azafullerene C_59_N

**DOI:** 10.3390/molecules29030609

**Published:** 2024-01-26

**Authors:** Xiong Li, Shuyi Wang, Jingdong Guo, Ziye Wu, Changrui Guo, Shaohong Cai, Mingsen Deng

**Affiliations:** 1 College of Big Data and Information Engineering, Guizhou University, Guiyang 550025, China; lix@ecut.edu.cn (X.L.); shuyiwang@gznc.edu.cn (S.W.); 2 School of Science, East China University of Technology, Nanchang 330013, China; 3 Guizhou Provincial Key Laboratory of Computational Nano-Material Science, Guizhou Education University, Guiyang 550018, China; jingdongguo@126.com; 4 School of Information, Guizhou University of Finance and Economics, Guiyang 550025, China; zywu@mail.gufe.edu.cn (Z.W.); guochangrui@mail.gufe.edu.cn (C.G.); 5 Department of Resources and Environment, Moutai Institute, Renhuai 564507, China

**Keywords:** azafullerene, oxide, hydrate, NEXAFS, XES, XPS shake-up

## Abstract

The interaction of fullerenes and their derivatives with environmental molecules such as oxygen or water was crucial for the rational design of low-dimensional materials and devices. In this paper, the near-edge X-ray absorption fine structure (NEXAFS), X-ray emission spectroscopy (XES) and X-ray photoelectron spectroscopy (XPS) shake-up satellites were employed to distinguish the oxides and hydrates of the fullerene C_60_ and azafullerene C_59_N families. The study includes various isomers, such as the open [5,6] and closed [6,6] isomers of C_60_O, C_60_H(OH), C_60_-O-C_60_, C_60_H-O-C_60_H, C_59_N(OH) and C_59_N-O-C_59_N, based on density functional theory. These soft X-ray spectra offered comprehensive insights into the molecular orbitals of these azafullerene molecular groups. The oxygen *K*-edge NEXAFS, carbon and oxygen *K*-edge XPS shake-up satellite spectra provided valuable tools for distinguishing oxides or hydrates of fullerene C_60_ and azafullerene C_59_N. Our findings could significantly benefit the development of fullerene functional molecular materials and expand the application scope of soft X-ray spectroscopy as a molecular fingerprinting tool for the fullerene family.

## 1. Introduction

The carbon allotrope fullerene C_60_ and its molecular crystals are considered fundamental units in probing the mechanisms of transport and photoelectric processes. Their fast carrier mobility and strong electron affinity position fullerenes are at the forefront of electronic device applications, including field-effect transistors [1] or photovoltaics [2], nanomedicine [3], nanoelectronics [4], organic and inorganic nanocomposites [5,6]. The azafullerene C_59_N monomer, notable for its unpaired electrons and resultant free radical behavior [7], remains a vibrant research area despite its instability. For example, the report of Martín-Gomis et al. showed promising implications in the development of organic solar cells built with covalent donor-acceptor systems using C_59_N as the acceptor [8]. Further, the C_59_N system has been shown to achieve a more persistent charge-separated state [9], underscoring its potential in advanced applications.

However, the insertion of extrinsic species, such as oxygen and water, could substantially alter the physical properties of these electronic materials [10]. Numerous studies have reported evidence for the sensitivity of fullerene or azafullerene to the incorporation of such impurities [11,12,13,14]. To further understand these impacts, Lin et al. conducted a detailed study on the oxidation process of azafullerene C_59_N [15]. Research has established that oxides and hydrates of fullerene and azafullerene are key materials in constructing a variety of fullerene-based nanostructures [16,17]. As a straightforward derivative of fullerene, C_60_O has emerged as a foundational material for producing more complex fullerene oxides, playing a crucial role in the synthesis of diverse compounds [18,19,20]. Fedurco et al. and Jones et al. demonstrated that N@C_60_O represented a valuable intermediary for researchers engaged in quantum information processing at the molecular level [21]. The work of Mondal et al. has also highlighted the complex interplay between thermal and mechanical transformations within the C_60_O cluster, which could influence its thermoelectric properties through the modulation of intermolecular interactions [22]. Moreover, the C_59_N oxides have been identified as robust recyclable catalysts, featuring a rate-determining energy barrier of 0.80 eV [23].

The presence of oxides and water-related reactions in fullerene and azafullerene compounds often leads to substantial modifications in the physical properties of organic semiconductors. These modifications are instrumental in the fabrication of numerous fullerene nanostructures. However, the properties of the oxides and hydrates exhibit significant variations [18]. Consequently, understanding their electronic and chemical characteristics is essential for further exploration and utilization. In this regard, Palotás et al. have made a noteworthy contribution by providing the initial experimental infrared multiple-photon dissociation vibrational spectra of gaseous C_60_O^+^ and C_60_OH^+^. However, challenges persist, as the experimental band centered at 1333 cm^−1^ was not accurately reproduced by the theoretical [6,6]C_60_OH^+^ spectrum [24]. This discrepancy demands considerable efforts dedicated to acquiring a deeper understanding of these materials, as evident in the studies mentioned earlier [25,26,27,28], where X-ray spectra have proven to be valuable experimental methods for investigating the electronic structures of aza[60]fullerene [29,30,31].

Near-edge X-ray absorption fine structure (NEXAFS) is a well-established spectroscopic approach employed for examining the local molecular structure and acquiring insights into unoccupied molecular orbitals. X-ray emission spectroscopy (XES) was frequently utilized to investigate the occupancy density of molecular electronic states. Both NEXAFS and XES offered element and site-specific capabilities, making them potent tools for elucidating intricate electronic properties of materials. Additionally, X-ray photoelectron spectroscopy (XPS) shake-up satellites, engendered by excitations of valence electrons concomitant with the ionization of core electrons, provided information regarding the valence band of materials in the vicinity of the core-hole screening [32,33,34,35,36,37,38,39]. For instance, Klein et al. utilized the delta-self-consistent-field (ΔSCF) and transition potential (TP) methods for calculating XPS and NEXAFS transitions, allowing the prediction of *K*-shell information [40]. Meanwhile, Armillotta et al. investigated the mechanisms of metal intermolecular coordination using NEXAFS technology [41].

**Figure 1:**
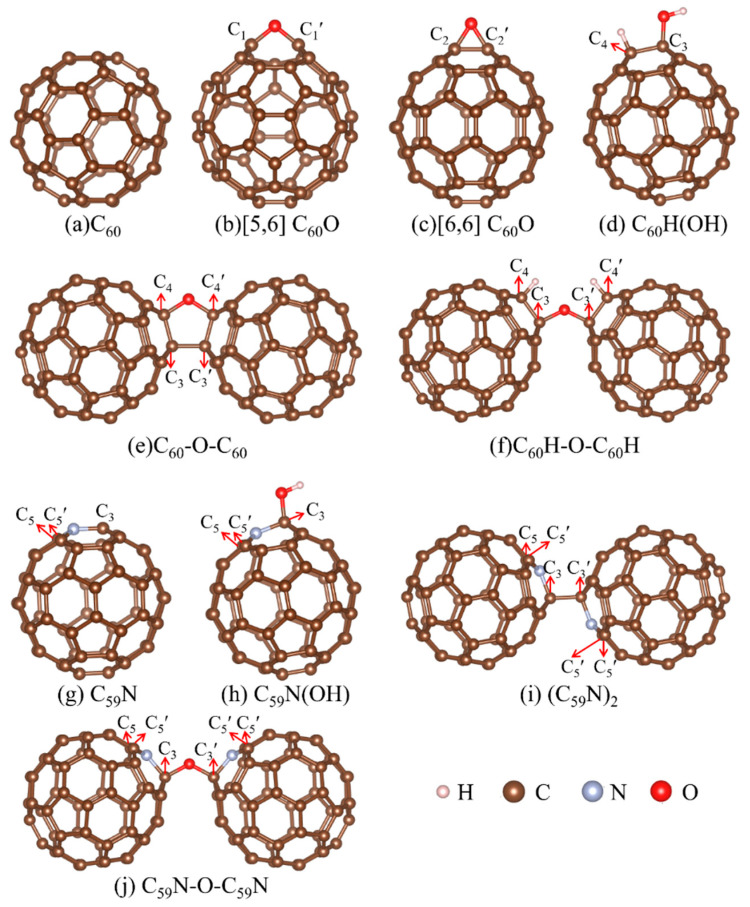
The optimized molecular models of the fullerene C_60_, aza[60]fullerene C_59_N, their oxides, and hydrates, the carbon atoms C_1_ and C_1_′, C_2_ and C_2_′, C_3_ and C_3_′, C_4_ and C_4_′ and C_5_ and C_5_′ are symmetrically equivalent.

The open [5,6] and closed [6,6] isomers of C_60_O, as well as C_60_-O-C_60_, are examples of molecular crystals that had been synthesized [42,43,44]. Creegan et al. identified the epoxide isomer of C_60_O through ^13^C NMR and FTIR examinations, revealing that the oxygen atom bridges to carbon atoms of two hexagons with *C_2v_* symmetry [42]. Weisman et al. further demonstrated that C_60_O can be prepared in both closed [6,6] epoxide and open [5,6] annulene isomers, depending on the synthesis method [44]. The C_60_-O-C_60_ molecular crystal consists of a C_60_ dimer, and an oxygen atom bridged in a furan-like ring (Figure 1e) [43], while the two isomers of C_60_O are composed of a C_60_ monomer and an oxygen atom in the form of epoxide and annulene, respectively (Figure 1b,c) [42]. Erbahar et al. discovered that the most stable structure for a single oxygen atom within (C_59_N)_2_ involves the oxygen atom in an ether configuration, forming a cross-linking bond between two (C_59_N) units [45], namely C_59_N-O-C_59_N (Figure 1j). In the reaction between H_2_O and C_60_, the configuration of the cross-linking bond could also be considered, specifically C_60_H-O-C_60_H (Figure 1f). In this study, the NEXAFS, XES and XPS shake-up satellites for seven exemplary members of the hydrates and oxides of fullerene C_60_ and aza[60]fullerene C_59_N, namely open [5,6] and closed [6,6] isomers of C_60_O, C_60_H(OH), C_60_-O-C_60_, C_60_H-O-C_60_H, C_59_N(OH) and C_59_N-O-C_59_N, were studied by employing the full core-hole (FCH) potential method as well as equivalent core-hole Kohn–Sham (ECH-KS) approach. The study provides valuable insights that can support and guide further experimental investigations related to these oxide materials and water-involved reactions.

## 2. Results and Discussion

The optimized molecular models of the fullerene C_60_, aza[60]fullerene C_59_N, their oxides and hydrates are shown in Figure 1. The carbon atoms C_1_ and C_1_′, C_2_ and C_2_′, C_3_ and C_3_′, C_4_ and C_4_′ and C_5_ and C_5_′ are symmetrically equivalent. The binding energies (BE) of the H atom, O atom, OH, C_59_N, and C_60_ to the C_59_N and C_60_ monomers were calculated to obtain the stability of oxides and hydrates of the fullerenes and azafullerenes by

(1) $$E_{BE}=E_{tot}-\sum_{i=1}^{n}E_{i}$$

where $E_{tot}$ is the electronic energy of C_60_, open [5,6] and closed [6,6] isomers of C_60_O, C_60_H(OH), C_60_-O-C_60_, C_60_H-O-C_60_H, C_59_N, C_59_N(OH), (C_59_N)_2_ and C_59_N-O-C_59_N molecules, *E_i_* is the electronic energy of the isolated H atom, O atom, OH, C_59_N and C_60_ molecule, which are the monomers that constitute the ten molecules mentioned above. HOMO-LUMO energy gap (*E*_gap_) is calculated by

(2) $$E_{gap}=E_{LUMO}-E_{HOMO}$$

where $E_{LUMO}$ are LUMO energies and $E_{HOMO}$ are HOMO energies. The binding energies, HOMO and LUMO energies and *E*_gap_ are collected in Table 1, which are verified well with the theoretical investigations based on the B3LYP [46] and LDA-PW91 [45] level. The symmetry point groups and the number of symmetrically inequivalent carbon atoms for the ten molecule groups are also shown in Table 1. The energy gaps decrease upon oxidation and hydration of fullerene C_60_, whereas the gaps increase for azafullerene C_59_N. The energy gap change trends indicated that the fullerene C_60_ becomes more reactive after oxidation and hydration, while azafullerene C_59_N becomes less reactive after oxidation and hydration. The binding energies confirm that these oxides and hydrates are more stable than the (C_59_N)_2_ molecule, with the C_60_H-O-C_60_H molecule identified as the most stable oxidation configuration.

The HOMOs and LUMOs of the ten molecular groups, calculated with B3LYP/6-311+G*, are presented in Figure S1, which are plotted with VESTA [47]. The isovalues for C_60_-O-C_60_, C_60_H-O-C_60_H, (C_59_N)_2_ and C_59_N-O-C_59_N molecules were set to 0.02, while that of the other six molecular groups was set to 0.04. The calculated energy of HOMO and LUMO of C_60_, C_59_N and (C_59_N)_2_ are in good agreement with the works of Mohammadi and colleagues [48,49,50]. It is notable that the two isomers of C_60_O had nearly the same energy gap and only a 2.11 kcal/mol difference in binding energy. Yet, they exhibit distinct electronic features, such as in O_3_ adsorption [18].

### 2.1. Carbon K-edge NEXAFS and XES

**Figure 2:**
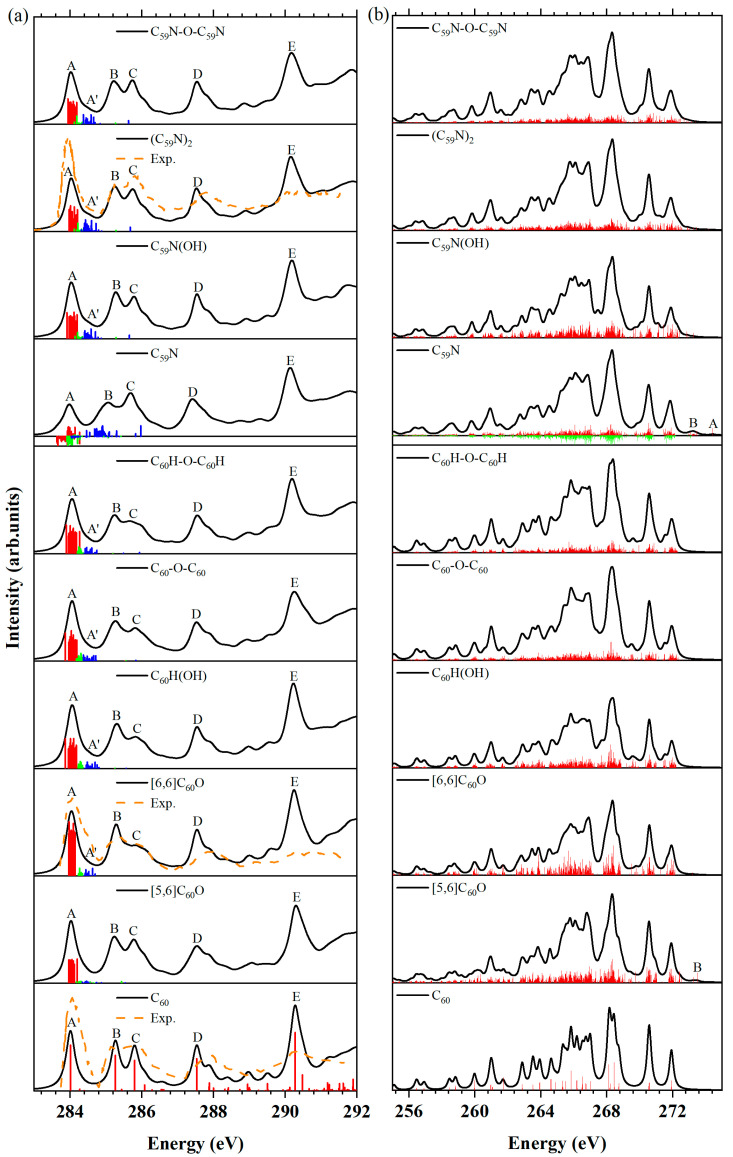
(**a**) NEXAFS spectra at the carbon *K*-edge of the fullerene C_60_, aza[60]fullerene C_59_N, their oxides and hydrates and (C_59_N)_2_. The orbital transitions from the 1*s* to the LUMO (shown as red bars), LUMO + 1 (shown as green bars) and LUMO + 2 (shown as blue bars) in the NEXAFS spectra of the nine molecules, along with the discrete intensities (shown as red bars) of the NEXAFS spectrum of the fullerene C_60_ molecule were plotted below each convoluted spectrum. For the C_59_N molecule, the contributions from β-spin electrons were marked as negative numbers. Experimental NEXAFS spectra of C_60_, [6,6]C_60_O [51] and (C_59_N)_2_ molecules [52] have been shifted −0.5 eV (shown as dashed lines). (**b**) Simulated XES spectra at the carbon *K*-edge of the fullerene C_60_, aza[60]fullerene C_59_N, their oxides and hydrates and (C_59_N)_2_. The discrete XES intensities of all molecules were depicted in the form of red bars (α-spin) and green bars (β-spin, represented as negative numbers) within the convoluted XES spectra.

The NEXAFS and XES spectra of carbon *K*-edges of the selected molecules, including the open [5,6] and closed [6,6] isomers of C_60_O, C_60_H(OH), C_60_-O-C_60_, C_60_H-O-C_60_H, C_59_N(OH) and C_59_N-O-C_59_N, were plotted in Figure 2. The spectra of the C_60_, C_59_N, and (C_59_N)_2_ molecules were also plotted for comparison. Also, the experimental NEXAFS spectra of C_60_, [6,6]C_60_O and (C_59_N)_2_ molecules we could find are reproduced from the work of Käämbre et al. [51] and Schulte et al. [52] for comparison, which are plotted in dashed lines. It is clear that simulated NEXAFS spectra of C_60_ and [6,6]C_60_O molecules are in good agreement with the experimental results. Our simulated spectra reproduced all the important characteristics of the experiment results.

Carbon *K*-edge NEXAFS. The NEXAFS spectra in Figure 2a exhibit five distinct peaks (labeled A–E) at nearly identical energy positions for each molecule, as illustrated in Figure 1. In addition, the orbital transitions from the 1*s* to the LUMO (shown as red bars), LUMO + 1 (shown as green bars) and LUMO + 2 (shown as blue bars) in the NEXAFS spectra of the nine molecules, along with the discrete intensities (shown as red bars) of the NEXAFS spectrum of the fullerene C_60_ molecule were plotted below each convoluted spectrum. For the C_59_N molecule, the contributions from β-spin electrons are represented as negative numbers. Although the NEXAFS spectra of these ten molecules exhibit similar peak positions, some notable differences can be observed. Specifically, depending on their lower symmetry—*C_2h_* for (C_59_N)_2_, *C_2v_* for closed [6,6] isomer of C_60_O, C_60_-O-C_60_, C_60_H-O-C_60_H, C_59_N-O-C_59_N and *Cs* for open [5,6] isomer of C_60_O, C_60_H(OH) and C_59_N(OH), the NEXAFS spectra of these molecules exhibit a larger number of molecular orbital (MO) transitions from C 1*s* orbitals to unoccupied orbitals within each peak in comparison to that of C_60_. From the feature peak E to their low-energy region, which corresponds to the C *K*-shell 1*s* absorption edges, signals that the C *K*-shell 1*s* absorption edges of all ten molecules are nearly identical. This indicated that both fullerene C_60_ and aza[60]fullerene C_59_N maintain the same valence state of carbon even after oxidation or exposure to water.

In the oxides and hydrates of fullerene C_60_, the NEXAFS spectrum of the closed [6,6] isomer of C_60_O resembles that of the C_60_H(OH) and C_60_H-O-C_60_H, as they all have a relatively weak and broad peak C and a strong peak B, and a very weak feature peak A’ between the peaks A and B can be observed. While peak B of the NEXAFS spectra of the other two molecules open the [5,6] isomer of C_60_O, and C_60_-O-C_60_ exhibit a slightly higher intensity than peak C, the feature peak A’ only traced in the C_60_-O-C_60_ molecule. The feature peak A’ primarily arose from the carbon 1*s* orbital to the LUMO + 2. Notably, the transition 1*s* → LUMO + 2 is notably weaker in the open [5,6] isomer of C_60_O. The feature peak A’ is linked to the change of electronic structures of the C_60_ molecule upon its interaction with another atom or molecule.

It is evident that the NEXAFS spectra for C_59_N(OH), (C_59_N)_2_ and C_59_N-O-C_59_N molecules all have a weak feature peak A′, which is the marker of the oxides or reactions involving H_2_O of aza[60]fullerene C_59_N. However, they exhibit significant differences in comparison to that of the C_59_N molecule, which lacks peak A′. Remarkably, nearly all carbon atoms exhibit similar NEXAFS spectral feature peaks A, B, C, D and E, regardless of any specific molecule. However, there is one notable exception, where feature peak A lacks contribution from the nearest carbon atoms (C_1_, C_2_, C_3_, C_4_, C_5_ in Figure 1) neighboring the nitrogen and oxygen atoms. This distinction in feature peak A among the spectra of the neighbor of the nitrogen, oxygen and other carbon atoms could be attributed to their distinct local (chemical) environments.

Carbon *K*-edge XES. The XES spectra at the carbon *K*-edge of the ten molecules were plotted in Figure 2b. The discrete XES intensities of [5,6]C_60_O and C_59_N are shown in red bars (α-spin) and green bars (β-spin). These bars are superimposed on their convoluted XES spectra. The XES spectra of these ten molecules exhibit limited discernible differences in contrast to the NEXAFS spectra, except for specific peaks. The peak A in the C_59_N molecule can be attributed to the MO transition from the HOMO to the 1*s* orbital of carbon atom C_3_ [46], and the peak B can be attributed to the transitions from the HOMO to the 1*s* orbitals of other carbon atoms. Similarly, peak B in the [5,6]C_60_O isomer primarily arises from the MO transitions between the HOMO and the 1*s* orbital of the carbon atoms. Consequently, it might be beneficial to utilize the feature peak B in the XES spectrum of the [5,6]C_60_O isomer to examine its presence within C_60_. Although the experimental aspects might pose certain challenges, employing these distinctive features can provide valuable insights into their existence within the C_60_ crystalline and provide effective evidence for distinguishing the open [5,6] isomers of C_60_O from oxides and hydrates of C_60_.

In summary, the carbon *K*-edge NEXAFS and XES spectra provide electronic structure information about the oxides and hydrates of C_60_ and C_59_N, which can be used to distinguish the open [5,6] isomer of C_60_O. However, distinguishing other oxides and hydrates using these spectra remains challenging.

### 2.2. Nitrogen K-edge NEXAFS and XES

**Figure 3:**
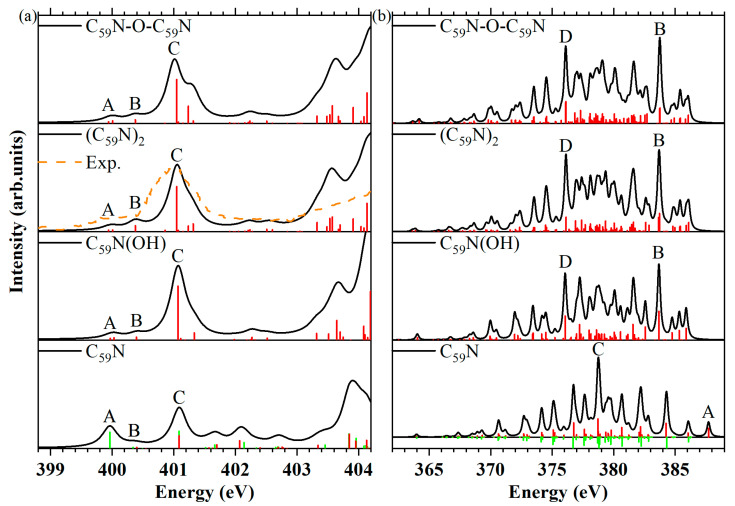
(**a**) Simulated NEXAFS spectra at the nitrogen *K*-edge of C_59_N, C_59_N(OH), (C_59_N)_2_ and C_59_N-O-C_59_N molecules, discrete intensities before convolution were represented by red bars for α-spin and green bars for β-spin. The experimental NEXAFS spectra of (C_59_N)_2_ molecule [52] are shown in dashed lines. (**b**) Simulated XES spectra at the nitrogen *K*-edge of these four molecules, discrete intensities of XES of all molecules were in red (α-spin) and green bars (β-spin, represented as negative numbers) within the convoluted XES spectra.

To detect the presence of C_59_N(OH) and C_59_N-O-C_59_N, which are the oxides or hydrates of aza[60]fullerene C_59_N, the nitrogen *K*-edge NEXAFS and XES spectra were simulated, as shown in Figure 3. The spectra of the C_59_N and (C_59_N)_2_ were also plotted for comparison. The experimental NEXAFS spectrum of (C_59_N)_2_ molecule is shown in dashed lines reproduced from the work of Schulte and colleagues [52]. It can be found that the simulated and experimental spectra are in good agreement.

Nitrogen *K*-edge NEXAFS. Both the C_59_N(OH) and C_59_N-O-C_59_N exhibit a faint shoulder A in the NEXAFS spectra (Figure 3a), which is attributed to the nitrogen 1*s* to LUMO transition. In contrast, the C_59_N molecule presents a relatively more pronounced first peak (peak A) at lower excitation energy, resulting from the nitrogen 1*s* → LUMO transition of β-spin. The MO transitions and corresponding energies of peak or shoulder A are available in Table S1. Despite the significance of the core-hole effect in comprehending NEXAFS spectra, the disparity observed in the nitrogen 1*s* → LUMO MO transition among the four molecules could be elucidated by considering the distribution of the LUMOs in the Ground state (GS). The LUMOs of C_59_N(β-spin), C_59_N(OH), (C_59_N)_2_ and C_59_N-O-C_59_N were plotted in Figure S1g–j, respectively. The LUMO of C_59_N exhibits a noticeable distribution around the nitrogen atom, whereas the LUMO of C_59_N(OH) exhibits a pronounced carbon character, similar to the (C_59_N)_2_, which fits well with prior results [53,54]. The LUMO in the C_59_N-O-C_59_N molecule is predominantly concentrated on the intermolecular bond of carbon, which contributes to the existence of a weak shoulder A in their NEXAFS spectra. Notably, the weak shoulder A observed in C_59_N(OH) and C_59_N-O-C_59_N are valuable for distinguishing them from C_59_N.

Nitrogen *K*-edge XES. The XES spectra at the nitrogen *K*-edge provide valuable insights, particularly when combining information obtained from GS calculations. In the case of the C_59_N(OH), (C_59_N)_2_ and C_59_N-O-C_59_N, their HOMOs exhibit some nitrogen characteristics, as illustrated in Figure S1h–j. For those compounds, the transitions from the HOMO to the nitrogen 1*s* orbital occur at relatively lower energies compared with the energy corresponding to the distinct peak A in the C_59_N molecule. The peak A in C_59_N originates from the transition from the HOMO (α-spin) to nitrogen 1s orbital transition [46]. Interestingly, the XES spectra of both C_59_N(OH) and C_59_N-O-C_59_N exhibit strong and sharp feature peaks B and D, resembling those observed in (C_59_N)_2_. This similarity is in stark contrast to the sharp and pronounced feature, peak C, observed in the C_59_N. These peaks are a result of transitions originating from deeper valence orbitals to the nitrogen 1*s* orbital(s), allowing for a clear distinction between C_59_N(OH) and C_59_N-O-C_59_N when compared with C_59_N.

### 2.3. Oxygen K-edge NEXAFS and XES

**Figure 4:**
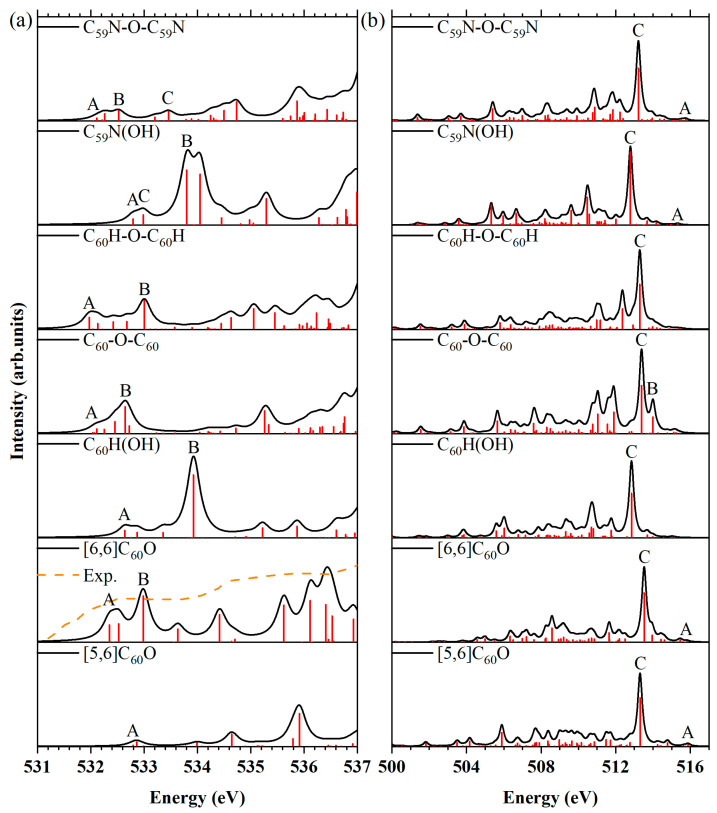
(**a**) Simulated NEXAFS spectra at the oxygen *K*-edge of the open [5,6] and closed [6,6] isomers of C_60_O, C_60_H(OH), C_60_-O-C_60_, C_60_H-O-C_60_H, C_59_N(OH) and C_59_N-O-C_59_N, discrete intensities before convolution were in red bars. The experimental NEXAFS spectrum of [6,6]C_60_O molecule [55] is shown in dashed lines. (**b**) Simulated XES spectra at the oxygen *K*-edge of these seven oxygen-containing molecules, discrete intensities of XES of all molecules were in red bars within the convoluted XES spectra.

Oxygen *K*-edge NEXAFS. The simulated NEXAFS spectra at the oxygen *K*-edge of the seven oxygen-containing molecules are shown in Figure 4a. The experimental NEXAFS spectrum of [6,6]C_60_O molecule is shown in the dashed line, which is reproduced from the work of Wohlers et al. at 370K [55]. The energy positions of peak (shoulder) A (corresponds to 1s → LUMO molecular orbital transition) and peaks B and C are listed in Table 2. Both the [6,6]C_60_O and the C_60_H-O-C_60_H produce the first shoulder A or peak A with moderate intensity, which arises from the transition of oxygen 1*s* to the LUMO. The peak A of the NEXAFS spectra of the closed [6,6] isomer of C_60_O is observed at a higher excitation energy (532.35 eV), whereas it appears at a lower excitation energy in the C_60_H-O-C_60_H (531.97 eV). Conversely, the oxygen 1*s* → LUMO transition only produces a weak peak A in the C_60_H(OH) and a weak shoulder A in the [5,6]C_60_O, C_60_-O-C_60_, C_59_N(OH) and C_59_N-O-C_59_N molecules. The disparity observed in the oxygen 1*s* → LUMO transition among these seven molecules could also be elucidated by examining the distribution of their LUMOs in the GS. Unlike the [5,6]C_60_O, C_60_H(OH), C_60_-O-C_60_, C_59_N(OH) and C_59_N-O-C_59_N, whose LUMOs have strong carbon character (Figure S1b,d,e,h,j), the LUMOs of the [6,6]C_60_O and the C_60_H-O-C_60_H molecules present here also exhibit some distribution around the oxygen atom (Figure S1c,f). Therefore, their NEXAFS spectra exhibit a moderate shoulder A or peak A. In particular, the moderately strong shoulder A with its relatively high excitation energy in the [6,6]C_60_O and the moderately strong peak A with its relatively low excitation energy in the C_60_H-O-C_60_H can be considered as the fingerprint of their existence.

Furthermore, in the case of the [5,6]C_60_O, C_60_H(OH) and C_59_N(OH), the oxygen 1*s* → LUMO transition is shown in the initial peak A or shoulder A with a weak intensity at nearly identical higher excitation energies (532.80 eV, 532.64 eV and 532.78 eV), among the five molecules with pronounced carbon characteristics in their LUMOs. The molecular groups, including the open [5,6] isomer of C_60_O, C_60_H(OH) and C_59_N(OH), show that the absence of a strong peak B in comparison to the other two molecules, which can serve as the fingerprint of [5,6]C_60_O. Additionally, C_60_H(OH) can be distinguished by the presence of a distinct peak B, while the presence of C_59_N(OH) can be recognized by the strong peak B and weak peak C. The spectra of the other two molecules, C_60_-O-C_60_ and C_59_N-O-C_59_N, fall into another category, in which the oxygen 1*s* → LUMO transition generates the initial shoulder A with a weak intensity at nearly identical lower excitation energies (532.03 eV and 532.04 eV). These could be attributed to the influence of another carbon cage. At the same time, the energy positions of their peak B are also quite similar (532.64 eV and 532.51 eV). Nevertheless, the distinct feature peak C at 533.45 eV in the C_59_N-O-C_59_N molecule serves as its distinguishing characteristic compared with C_60_-O-C_60_.

Oxygen *K*-edge XES. The XES spectra at the oxygen *K*-edge are shown in Figure 4b. The MO transitions and corresponding energies of peak A are compared in Table S2. The XES spectrum of the [5,6] and [6,6] isomers of C_60_O, as well as the C_59_N-O-C_59_N, exhibit a discernible weak peak A at high energy (515.88 eV, 515.82 eV and 515.74 eV). These peaks arise from the HOMO to oxygen 1*s* orbital transition. The HOMOs of the [5,6] and [6,6] isomers of C_60_O and C_59_N-O-C_59_N, as depicted in Figure S1b,c,j, exhibit some characteristics of oxygen. This results in the manifestation of the moderate peak A in their XES spectra. Conversely, the HOMOs of C_60_H(OH), C_60_-O-C_60_ and C_60_H-O-C_60_H possess strong carbon attributes, as shown in Figure S1d–f. The transition from the HOMO to the oxygen 1*s* orbital does not result in the observation of any discernible peaks in their XES spectra. For the C_59_N(OH), although its HOMOs also exhibit little oxygen characteristics, the transition from the HOMO to the oxygen 1*s* orbital occurs at relatively lower energy (515.33 eV) compared with the [5,6] and [6,6] isomers of C_60_O, as well as C_59_N-O-C_59_N. Significantly, the XES spectra reveal distinct characteristics, including the moderately strong peak B in the C_60_-O-C_60_, as well as the uniformly strong and sharp peak C in all seven molecules. These peaks arise from transitions originating in the deeper valence orbitals to the oxygen 1*s* orbital, allowing for the distinction of oxides or hydrates of different fullerene and azafullerene.

### 2.4. XPS Shake-Up Satellites

**Figure 5:**
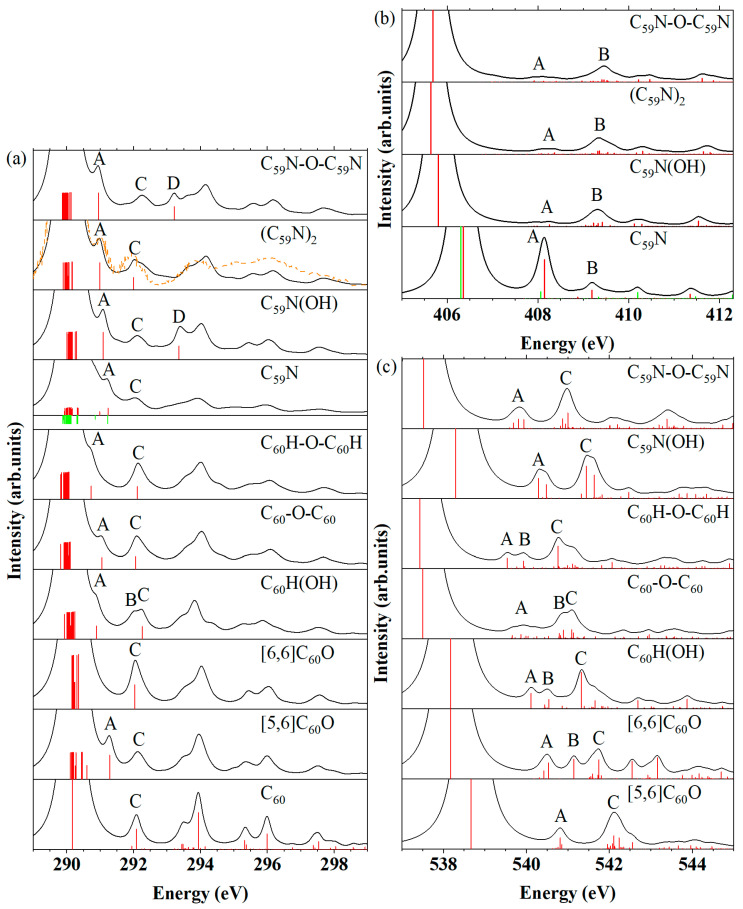
(**a**) XPS shake-up satellites at the carbon *K*-edge for the fullerene C_60_, aza[60]fullerene C_59_N, their oxides and hydrates and (C_59_N)_2_, along with the discrete intensities of the C_60_ molecule before convolution were depicted as red bars. The XPS main lines of all symmetrically inequivalent carbon atoms are represented as red bars underneath each XPS shake-up spectrum of the other nine members. Contributions from the β-spin electrons in the C_59_N molecule are shown as green bars. The experimental XPS shake-up satellite spectrum of (C_59_N)_2_ molecule [52] is shown as dashed lines. (**b**) XPS shake-up satellites at the nitrogen *K*-edge for C_59_N, C_59_N(OH), (C_59_N)_2_ and C_59_N-O-C_59_N molecules, along with the discrete intensities before convolution, are depicted as red bars. Contributions from the β-spin electrons are also shown as green bars under the spectrum in the C_59_N molecule. (**c**) XPS shake-up satellites at the oxygen *K*-edge for the open [5,6] and closed [6,6] isomers of C_60_O, C_60_H(OH), C_60_-O-C_60_, C_60_H-O-C_60_H, C_59_N(OH) and C_59_N-O-C_59_N, along with the discrete intensities before convolution are shown as red bars.

Carbon *K*-edge XPS shake-up satellites. The calculated XPS shake-up satellites at the carbon *K*-edge for the fullerene C_60_, aza[60]fullerene C_59_N, their oxides and reactions involving H_2_O and (C_59_N)_2_ were plotted in Figure 5a. Additionally, spectra of C_60_, C_59_N and (C_59_N)_2_ were also provided for comparison. The XPS main lines of all symmetrically inequivalent carbon atoms are represented as red bars underneath each XPS shake-up spectrum of the other nine members. Contributions from the β-spin electrons in the C_59_N molecule were plotted as green bars. The experimental XPS shake-up satellite spectrum of (C_59_N)_2_ molecule is reproduced from the work of Schulte et al. [52], which is in agreement very well with our simulated spectrum. It is evident that the XPS shake-up satellites of the oxides or hydrates of fullerene C_60_ show significantly different profiles compared with those of C_60_, except for that of [6,6] C_60_O. This observation signifies an alteration in the electronic structures of the valence orbitals following the oxidation or reaction with water of the C_60_ molecule. Similarly, the spectra of the C_59_N(OH) and C_59_N-O-C_59_N exhibit distinct profiles compared with those of the C_59_N and aza[60]fullerene dimer (C_59_N)_2_.

The spectra of these ten molecules exhibit very similar profiles, featuring prominent peaks A and C located proximate to the XPS main line, except for C_60_ and [6,6]C_60_O. The presence of shoulder B can be attributed to contributions from several carbon atoms in C_60_H(OH). The primary contributions to the peaks A and C in the nine molecules have been elucidated and are collected in Table 3. It is interesting that except for the peak C in the open [5,6] isomer of C_60_O, C_59_N, C_59_N(OH) and C_59_N-O-C_59_N, resulting from the shake-up mechanisms, the primary contributions to the peaks A and C in the other five molecules are actually the XPS main lines of the atoms C_1_, C_2_, C_3_, C_4_ and C_5_. It is evident that the carbon atom C_1_ (*sp*^2^-like) is connected to the oxygen atom, C_3_ (in the C_59_N) and C_5_ (in the C_59_N, C_59_N(OH), (C_59_N)_2_ and C_59_N-O-C_59_N) are bonded to the nitrogen atom with *sp*^2^-like, while the carbon atoms C_2_ (connected to the oxygen atom), C_3_ (in the C_60_H(OH), C_60_-O-C_60_, C_60_H-O-C_60_H, C_59_N(OH), (C_59_N)_2_, and C_59_N-O-C_59_N) and C_4_ (in the C_60_H(OH), C_60_-O-C_60_, and C_60_H-O-C_60_H) are *sp*^3^-like atoms in the closed [6,6] isomer of C_60_O, C_60_H(OH), C_60_-O-C_60_, C_60_H-O-C_60_H, C_59_N(OH), (C_59_N)_2_ and C_59_N-O-C_59_N (Figure 1b–j). Hence, their binding energies exhibit a slight increase compared with those of other carbon atoms. The hybridization of carbon atoms and the blue-shifted binding energy of these hybridized carbon atoms relative to the XPS main line for the oxides or hydrates of fullerene C_60_ and aza[60]fullerene C_59_N are collected in Table 4.

The local electronic structures of the C_3_ and C_4_ atoms in the oxides or hydrates of fullerene C_60_ show minimal changes after bonding with other atoms (like H atom), such as (C_4_ in the C_60_H(OH) and C_4_ in the C_60_H-O-C_60_H), or a large molecule (like C_3_ in the C_60_-O-C_60_), although they become an *sp*^3^-like carbon, and consequently lead to blue-shifted binding energy—around 1 eV in the C_60_H(OH) (C_4_), C_60_-O-C_60_ (C_3_) and C_60_H-O-C_60_H (C_4_) in comparison to their XPS main line. In contrast, *sp*^3^-like carbon atoms bonded to oxygen atoms are shown a pronounced blue-shifted in their binding energy—approximately 2 eV in the closed [6,6] isomer of C_60_O(C_2_), C_60_H(OH) (C_3_), C_60_-O-C_60_ (C_4_) and C_60_H-O-C_60_H (C_3_) when compared with their XPS main line. The larger blue shifts demonstrated the local electronic structures of the *sp*^3^-like C_2_, C_3_ and C_4_ atoms changing a lot after being connected to an oxygen atom (such as C_2_ in the [6,6]C_60_O, C_3_ in the C_60_H(OH) and C_60_H-O-C_60_H, C_4_ in the C_60_-O-C_60_). The C *K*-edge XPS shake-up satellite spectra reveal the distinctive features of the [6,6]C_60_O molecule, characterized by a strong peak C and the absence of peak A, while C_60_H(OH) exhibits a unique shoulder B in its spectrum. Therefore, C *K*-edge XPS shake-up satellites serve as a valuable technique for detecting the presence of [6,6]C_60_O and C_60_H(OH) molecules, enabling their differentiation from other fullerene C_60_ oxides and hydrates.

In the oxides or hydrates of azafullerene C_59_N, the binding energy of carbon atom C_5_ is blue-shifted by approximately 1 eV relative to the XPS main line, while the binding energy of carbon atom C_3_ remains unchanged in the C_59_N molecule [46]. However, upon oxidation (e.g., C_59_N-O-C_59_N) or reaction with water (e.g., C_59_N(OH)), the local electronic structure of the carbon atom C_3_ undergoes significant alteration, transforming it into a *sp*^3^-like carbon atom in the C_59_N(OH) and C_59_N-O-C_59_N. Consequently, a substantial blue-shift binding energy of around 3 eV is observed in the C_59_N(OH) and C_59_N-O-C_59_N compared to their XPS main line, while the blue-shift is approximately 2 eV in the (C_59_N)_2_[46]. The unique peak D in the spectra of C_59_N(OH) and C_59_N-O-C_59_N molecules distinguish them from C_59_N and (C_59_N)_2_ molecules. However, the C *K*-edge XPS shake-up satellite spectra could not differentiate between C_59_N(OH) and C_59_N-O-C_59_N molecules.

It is noteworthy that only C_1_ exhibits a *sp*^2^-like hybridization, leading to a subtle blue shift in binding energy relative to the XPS main line among all the carbon atoms bonded to an oxygen atom. The uniqueness of the C_1_ atom demonstrates a remarkable change in the localized electronic structure of C_1_ in the [5,6]C_60_O isomer after breaking the five- and six-membered ring structures of the fullerene cage.

Nitrogen *K*-edge XPS shake-up satellites. The alterations in electronic structures can be elucidated through the analysis of nitrogen *K*-edge XPS shake-up satellites (Figure 5b). The energy of peak B in the spectra for the C_59_N, C_59_N(OH), (C_59_N)_2_ and C_59_N-O-C_59_N are shown in Table 5. In both C_59_N(OH) and C_59_N-O-C_59_N, peak A is relatively weaker compared with that of the C_59_N. Furthermore, peak B in C_59_N-O-C_59_N exhibits a blue shift of approximately 0.15 eV compared with peak B of the C_59_N(OH). An extensive assignment of the principal contributions to peaks A and B, which are observed in the nitrogen *K*-edge XPS shake-up satellites of these four members within the C_59_N family, is provided in Table S3. The information can serve as a valuable reference for future experimental investigations. It is evident that, apart from C_59_N and C_59_N(OH), feature B in the other two molecules is attributed to excitations originating from deeper valence orbitals.

Oxygen *K*-edge XPS shake-up satellites. The alterations in electronic structures can also be discerned from the oxygen *K*-edge XPS shake-up satellites, as depicted in Figure 5c. The energies of peaks A, B and C in the oxygen *K*-edge XPS shake-up satellites are shown in Table 6 for the seven oxygen-containing molecules. The [6,6]C_60_O, C_60_H(OH), C_60_-O-C_60_ and C_60_H-O-C_60_H exhibit three feature peaks A, B and C. The other three molecules, open [5,6] isomer of C_60_O, C_59_N(OH) and C_59_N-O-C_59_N, do not show a discernible peak B, highlighting a noticeable difference between the [6,6]C_60_O, C_60_H(OH), C_60_-O-C_60_ and C_60_H-O-C_60_H. In comparison to the [5,6]C_60_O molecule, the featured peak A of C_59_N(OH) demonstrates a red shift of 0.52 eV, whereas the characteristic peak A of the C_59_N-O-C_59_N exhibits a red shift of 1.00 eV. In contrast, in comparison with [6,6]C_60_O, the characteristic peak A of C_60_H(OH) shows a red shift of 0.42 eV, the feature peak A of the C_60_-O-C_60_ exhibits a red shift of 0.66 eV, and the characteristic peak A of the C_60_H-O-C_60_H shows a red shift of 1.00 eV. Hence, the oxygen *K*-edge XPS shake-up satellites offer a valuable technique to detect the presence of these seven molecules, which can also be used to distinguish them from other oxides or hydrates family members of fullerene C_60_ and aza[60]fullerene C_59_N.

The detailed assignments of the primary contributions to peaks A, B and C within the oxygen *K*-edge XPS shake-up satellites for these seven oxygen-containing molecules are collected in Table S4, which can provide a valuable reference for future experiments. It is notable that peak B includes excitations from deeper valence orbitals in C_60_-O-C_60_. With the exception of C_60_H(OH) and C_59_N(OH) molecules, peak C also encompasses excitations from deeper valence orbitals in the remaining five molecules.

## 3. Computational Details

The ten groups of molecular models are presented in Figure 1, including fullerene(C_60_), C_60_O with oxygen in the [5,6] open annulene and [6,6] closed epoxide isomers, C_60_H(OH), ether-oxygen-bridged fullerene dimer C_60_-O-C_60_, C_60_H-O-C_60_H, aza[60]fullerene C_59_N, C_59_N(OH), (C_59_N)_2_ and C_59_N-O-C_59_N, in which C_60_, C_59_N and (C_59_N)_2_ molecules are used for comparison. All the molecular configurations were optimized, and the single point energy calculations were performed with the Gaussian16 program at the B3LYP/6-31G(d,p) level [56], and the optimized configurations were subsequently validated as energy minima through frequency calculations. All structures were optimized without initial symmetry constraints. The geometries with Cartesian coordinates of all optimized structures are listed in Table S7 The lowest frequencies of these structures are collected in Table S5, while the energies of the ten molecules with different spin multiplicities are collected in Table S6. The electronic state with the lowest energy was applied for the soft X-ray spectra simulations. Nitrogen, oxygen and their attached carbon atoms are shown in Figure 1b–j, where atoms C_1_ and C_1_′, C_2_ and C_2_′, C_3_ and C_3_′, C_4_ and C_4_′ and C_5_ and C_5_′ are symmetrically equivalent atoms.

The *Z* + 1 approximation and TP method have been employed for the efficient computation of large systems [57,58,59], which are characterized by a high number of MOs in the NEXAFS and XES spectroscopy calculations. To ensure computing precision, the FCH potential method was adopted for the accurate characterization of NEXAFS spectra [60,61,62,63,64]. The triple-ζ quality individual gauge for localized orbital (IGLO-Ⅲ) basis set was selected for the excited carbon, nitrogen or oxygen atoms, while other non-excited atoms were calculated by employing model core potentials (MCP), which was defined as the Huzinaga’s procedure [65] implemented in the StoBe program [66,67,68]. This computing strategy ensured the accuracy and speed for large-scale computing in our soft X-ray spectroscopy calculations [60,61]. Additionally, to enhance the accuracy, an augmented diffuse basis set (19s, 19p and 19d) for the excited carbon, nitrogen or oxygen atoms was utilized in the NEXAFS spectra calculations.

The NEXAFS spectra and ∆Kohn–Sham (∆KS) [60] calculations were carried out using StoBe [66,67,68]. The relativistic effects were accounted for by introducing energy shifts of +0.2 eV for the carbon *K*-edge, +0.3 eV for the nitrogen *K*-edge and +0.5 eV for the oxygen *K*-edge [69]. The NEXAFS spectra were generated by convoluting discrete intensities with a Lorentzian function, which incorporated spectral broadening. For energies below the ionization potential (IP), the full width at half maximum (FWHM) was set to 0.3 eV. In the range from the IP to 5 eV above, the FWHM increased linearly up to 1.0 eV. For energies beyond that range, a constant FWHM of 1.0 eV was employed. The XES spectra were simulated employing the BioNano-LEGO developed by Yi Luo’s group [70]. GS wave functions were derived from the Gaussian16 program [56] at the B3LYP/6-311+G* level. The computed XES spectra underwent additional convolution with a Lorentzian function, employing an FWHM of 0.3 eV.

The ECH-KS method was employed for XPS shake-up satellite spectra calculations [71,72,73], which has been introduced for the calculation of the numerous excited states necessary for the XPS shake-up satellites in large systems [74]. The 6-31G basis set was applied to the non-ionized atoms, while the IGLO-III basis set was utilized for the core-ionized carbon, nitrogen or oxygen atom [75]. The shake-up satellites were calculated by the ECH-KS method, which was consistent with ECH-TDDFT calculations and experimental results, as previously validated [75,76,77]. In our calculations, the XPS shake-up satellites were also performed by BioNano-LEGO [70]. The shake-up intensities were convolved with a Lorentzian function with an FWHM of 0.3 eV.

## 4. Conclusions

The core-hole excitation spectroscopic techniques, specifically NEXAFS, XES and XPS shake-up satellites, were employed to differentiate between various members of the oxides or hydrates family members of fullerene C_60_ and aza[60]fullerene C_59_N. These members include the open [5,6] and closed [6,6] isomers of C_60_O, C_60_H(OH), C_60_-O-C_60_, C_60_H-O-C_60_H, C_59_N(OH) and C_59_N-O-C_59_N. These spectra provided us with comprehensive insights into both the unoccupied and occupied molecular orbitals of the molecular groups under study. Meanwhile, the oxygen *K*-edge NEXAFS, carbon and oxygen *K*-edge XPS shake-up satellite spectra were employed to distinguish oxides or hydrates of fullerene C_60_ and azafullerene C_59_N. The approach allowed us to delve deeply insights into the relationships between individual spectral peaks and the electronic and geometric structures of the molecules within the fullerene C_60_ and aza[60]fullerene C_59_N families. These findings not only enhance our understanding of these complex molecular systems but also pave the way for future research and applications in the field of nanomaterial science.

## Figures and Tables

**Table 1:** Binding energies calculated using the B3LYP/6-31G(d,p) level, HOMO and LUMO energies, *E*_gap_, symmetry point groups and the number of symmetrically inequivalent carbon atoms for the fullerene C_60_, aza[60]fullerene C_59_N, their oxides and hydrates.

Molecule	HOMO Energy (eV)	LUMO Energy (eV)	*E*_gap_ (eV)	BE (kcal/mol)	Sym.	Number of Symmetrically Inequivalent Carbon
C_60_	−5.99	−3.23	2.76	–	*I_h_*	1
[5,6]C_60_O	−5.90	−3.30	2.60	−78.92	*C_s_*	32
[6,6]C_60_O	−5.94	−3.33	2.61	−76.81	*C_2v_*	16
C_60_H(OH)	−5.75	−3.19	2.56	−122.22	*C_s_*	32
C_60_-O-C_60_	−5.77	−3.31	2.46	−94.02	*C_2v_*	32
C_60_H-O-C_60_H	−5.71	−3.27	2.44	−220.17	*C_2v_*	32
C_59_N	−4.58 (α spin) −5.94 (β spin)	−3.29 (α spin) −3.46 (β spin)	1.29 (α spin) 2.48 (β spin)	–	*C_s_*	31
C_59_N(OH)	−5.62	−3.24	2.38	−72.53	*C_s_*	31
(C_59_N)_2_	−5.52	−3.29	2.23	−31.81	*C_2h_*	31
C_59_N-O-C_59_N	−5.55	−3.33	2.22	−123.60	*C_2v_*	31

**Table 2:** Energy of peak (shoulder) A, peaks B and C in the O *K*-edge NEXAFS for the [5,6] and [6,6] isomers of C_60_O, C_60_H(OH), C_60_-O-C_60_, C_60_H-O-C_60_H, C_59_N(OH) and C_59_N-O-C_59_N.

Molecule	Peak (Shoulder) A (eV) (1*s* → LUMO)	Peak B (eV)	Peak C (eV)
[5,6]C_60_O	532.80	−	−
[6,6]C_60_O	532.35	532.98	−
C_60_H(OH)	532.64	533.93	−
C_60_-O-C_60_	532.03	532.64	−
C_60_H-O-C_60_H	531.97	533.00	−
C_59_N(OH)	532.78	533.82	532.97
C_59_N-O-C_59_N	532.04	532.51	533.45

**Table 3:** The C *K*-edge XPS shake-up satellites of the fullerene C_60_, aza[60]fullerene C_59_N, their oxides and hydrates.

Molecule	A	C
[5,6]C_60_O	C_1_: 291.284 eV	… ^a^
[6,6]C_60_O	-	C_2_: 292.035 eV
C_60_H(OH)	C_4_: 290.888 eV	C_3_: 292.257 eV
C_60_-O-C_60_	C_3_: 291.055 eV	C_4_: 292.064 eV
C_60_H-O-C_60_H	C_4_: 290.735 eV	C_3_: 292.114 eV
C_59_N	C_5_: 291.245 eV (α spin)	… ^a^
	C_5_: 291.228 eV (β spin)	
C_59_N(OH)	C_5_: 291.096 eV	… ^a^
(C_59_N)_2_	C_5_: 290.989 eV	C_3_: 292.003 eV
C_59_N-O-C_59_N	C_5_: 290.952 eV	… ^a^

^a^ XPS shake-up satellites originating from multiple carbon atoms.

**Table 4:** The hybridization of carbon atoms and the blue-shifted binding energy of these hybridized carbon atoms relative to the XPS main line in the oxides or hydrates of fullerene C_60_ and aza[60]fullerene C_59_N.

Molecule	*sp*^2^-like Carbon Atom (Blue-Shift)	*sp*^3^-like Carbon Atom (Blue-Shift)
[5,6]C_60_O	C_1_ (∼1 eV)	-
[6,6]C_60_O	-	C_2_ (∼2 eV)
C_60_H(OH)	-	C_3_ (∼2 eV)
	-	C_4_ (∼1 eV)
C_60_-O-C_60_	-	C_3_ (∼1 eV)
	-	C_4_ (∼2 eV)
C_60_H-O-C_60_H	-	C_3_ (∼2 eV)
	-	C_4_ (∼1 eV)
C_59_N	C_3_ (–)	-
	C_5_ (∼1 eV)	-
C_59_N(OH)	C_5_ (∼1 eV)	C_3_ (∼3 eV)
(C_59_N)_2_	C_5_ (∼1 eV)	C_3_ (∼2 eV)
C_59_N-O-C_59_N	C_5_ (∼1 eV)	C_3_ (∼3 eV)

**Table 5:** Energy of peak B in the N *K*-edge XPS shake-up satellites for the C_59_N, C_59_N(OH), (C_59_N)_2_ and C_59_N-O-C_59_N molecules.

Molecule	Energy of Peak B (eV)
C_59_N	409.19
C_59_N(OH)	409.31
(C_59_N)_2_	409.34
C_59_N-O-C_59_N	409.46

**Table 6:** Energy of peaks A, B and C in the O *K*-edge XPS shake-up satellites for the [5,6] and [6,6] isomers of C_60_O, C_60_H(OH), C_60_-O-C_60_, C_60_H-O-C_60_H, C_59_N(OH) and C_59_N-O-C_59_N.

Molecule	Energy of Peak A (eV)	Energy of Peak (Shoulder) B (eV)	Energy of Peak C (eV)
[5,6]C_60_O	540.81	-	542.11
[6,6]C_60_O	540.53	541.14	541.75
C_60_H(OH)	540.11	540.54	541.33
C_60_-O-C_60_	539.87	540.89	541.10
C_60_H-O-C_60_H	539.53	539.94	540.76
C_59_N(OH)	540.29	-	541.44
C_59_N-O-C_59_N	539.81	-	541.00

## Data Availability

Data are contained within the article and Supplementary Materials.

## Supplementary Materials

The following supporting information can be downloaded at: https://www.mdpi.com/article/10.3390/molecules29030609/s1, Figure S1: The HOMOs and LUMOs of the fullerene C_60_, aza[60]fullerene, their oxides and reactions involving H2O calculated at B3LYP/6-311+G* level. The carbon atoms C_1_ and C_1_′, C_2_ and C_2_′, C_3_ and C_3_′ and C_4_ and C_4_′, which were connected to the oxygen atom, were symmetrically equivalent; Table S1: MO transitions and the corresponding energies for peak or shoulder A in the nitrogen K-edge NEXAFS of the C_59_N, C_59_N(OH), (C_59_N)2 and C_59_N-O-C_59_N molecules; Table S2: MO transitions and the corresponding energies for peak A in the oxygen K-edge XES of the open [5,6] and closed [6,6] isomers of C_60_O, C_59_N(OH) and C_59_N-O-C_59_N molecules; Table S3: Assignments of the nitrogen K-edge XPS shake-up satellites of the C_59_N, C_59_N(OH), (C_59_N)2 and C_59_N-O-C_59_N molecules; Table S4: Assignments of the oxygen K-edge XPS shake-up satellites of the open [5,6] and closed [6,6] isomers of C_60_O, C_60_H(OH), C_60_-O-C_60_, C_60_H-O-C_60_H, C_59_N(OH) and C_59_N-O-C_59_N molecules; Table S5: The lowest frequencies of all optimized structures; Table S6: The energies of the fullerene C_60_, aza[60]fullerene C_59_N, their oxides and hydrates molecules at different spin multiplicities; Table S7: Molecule coordinate of the open [5,6] and closed [6,6] isomers of C_60_O, C_60_H(OH), C_60_-O-C_60_, C_60_H-O-C_60_H, C_59_N(OH) and C_59_N-O-C_59_N molecules.
