# Supplementary material for: Core-Hole Excitation Spectra of the Oxides and Hydrates of Fullerene C60 and Azafullerene C59N
Source: Molecules. 2024 Jan 26;29(3):609. doi: 10.3390/molecules29030609 (PMC10856231; doi:10.3390/molecules29030609)
Supplement: Supplementary file 1 [file molecules-29-00609-s001.zip › molecules-2780190-supplementary.pdf]

Core-hole excitation spectra of the oxides and reactions involving water of fullerene C_{60} and azafullerene $C_{59}N$

Xiong Li [1,2], Shuyi Wang [1,3], Jingdong Guo [3], Ziye Wu [4], Changrui Guo [4], Shaohong Cai [4,5], *, Mingsen Deng [4], *

1 College of Big Data and Information Engineering, Guizhou University, Guiyang 550025, China

2 School of Science, East China University of Technology, Nanchang 330013, China

3 Guizhou Provincial Key Laboratory of Computational Nano-material Science, Guizhou Education University, Guiyang 550018, China

4 School of Information, Guizhou University of Finance and Economics, Guiyang 550025, China

5 Moutai Institute, Renhuai 564507, China

* Corresponding author: msdeng@mail.gufe.edu.cn(Mingsen Deng), caish@mail.gufe.edu.cn (Shaohong Cai)

Contents:

• Figure S1: The HOMOs and LUMOs of the fullerene C_{60}, aza[60]fullerene, their oxides, and reactions involving H_2O calculated at B3LYP/6-311+G* level. The carbon atoms C_1 and C_1', C_2 and C_2', C_3 and C_3', C_4 and C_4', which were connected to the oxygen atom, were symmetrically equivalent.

• Table S1: MO transitions and the corresponding energies for peak or shoulder A in the nitrogen *K*-edge NEXAFS of the $C_{59}N$, $C_{59}N(OH)$, $(C_{59}N)_2$, and $C_{59}N$-O-$C_{59}N$ molecules.

• Table S2: MO transitions and the corresponding energies for peak A in the oxygen *K*-edge XES of the open [5,6] and closed [6,6] isomers of $C_{60}O$, $C_{59}N(OH)$, and $C_{59}N$-O-$C_{59}N$ molecules.

• Table S3: Assignments of the nitrogen *K*-edge XPS shake-up satellites of the $C_{59}N$, $C_{59}N(OH)$, $(C_{59}N)_2$, and $C_{59}N$-O-$C_{59}N$ molecules.

• Table S4: Assignments of the oxygen *K*-edge XPS shake-up satellites of the open [5,6] and closed [6,6] isomers of $C_{60}O$, $C_{60}H(OH)$, C_{60}-O-C_{60}, $C_{60}H$-O-$C_{60}H$, $C_{59}N(OH)$, and $C_{59}N$-O-$C_{59}N$ molecules.

• Table S5: The lowest frequencies of all optimized structures.

• Table S6: The energies of the fullerene C_{60}, aza[60]fullerene $C_{59}N$, their oxides, and hydrates molecules at different spin multiplicities.

• Table S7: Molecules Coordinate of the open [5,6] and closed [6,6] isomers of $C_{60}O$, $C_{60}H(OH)$, C_{60}-O-C_{60}, $C_{60}H$-O-$C_{6}0H$, $C_{59}N(OH)$, and $C_{59}N$-O-$C_{59}N$ molecules.

A. Supplementary Figures

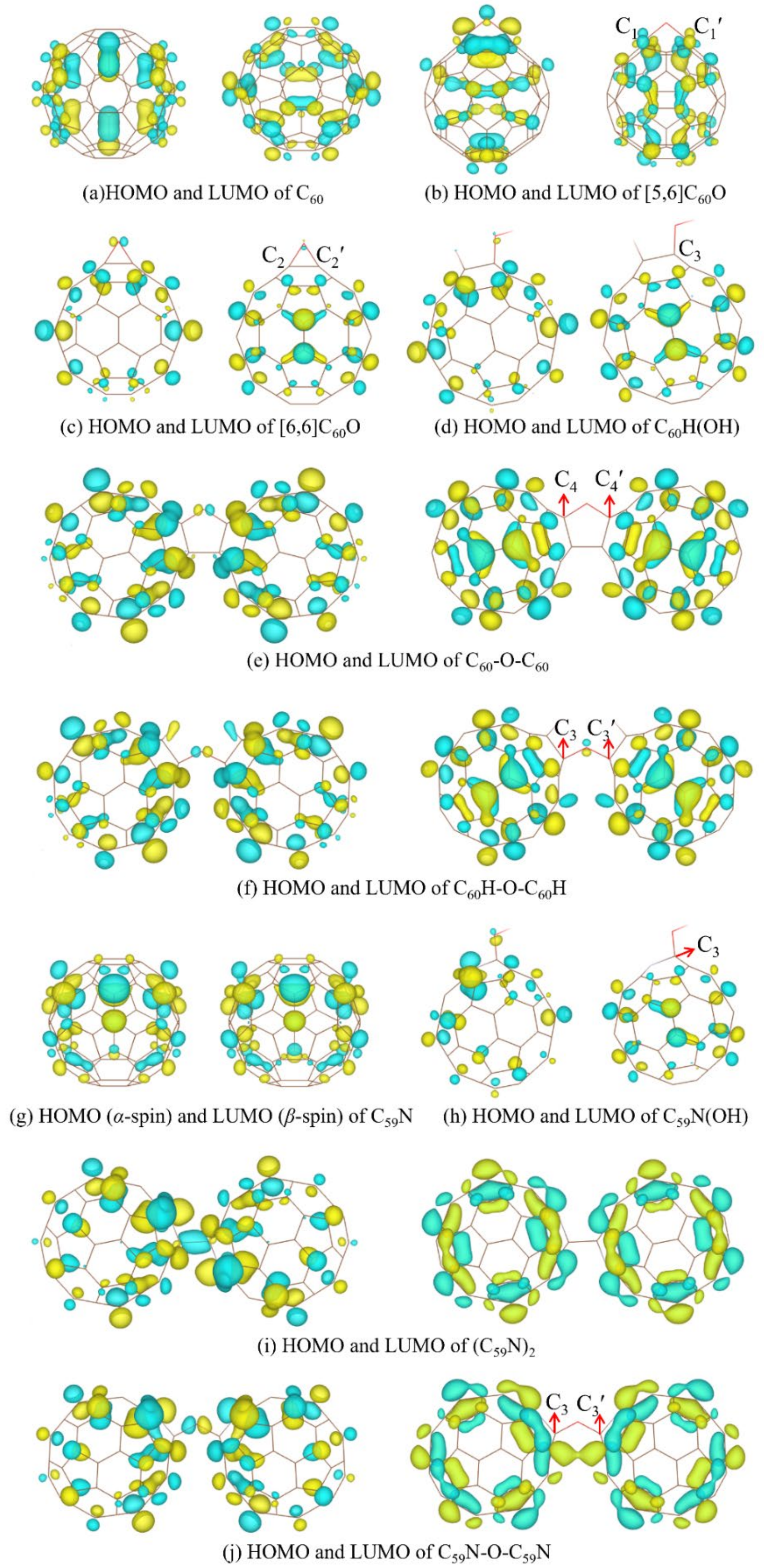

Figure S1. The HOMOs and LUMOs of the fullerene C_{60}, aza[60]fullerene, their oxides, and reactions involving H_2O calculated at B3LYP/6-311+G* level. The carbon atoms C_1 and C_1', C_2 and C_2', C_3 and C_3', C_4 and C_4', which were connected to the oxygen atom, were symmetrically equivalent.

B. Supplementary Tables

Table S1. MO transitions and the corresponding energies for peak or shoulder A in the nitrogen *K*-edge NEXAFS of the $C_{59}N$, $C_{59}N(OH)$, $(C_{59}N)_2$, and $C_{59}N$-O-$C_{59}N$ molecules.

**Molecule**	**MO transition of peak (shoulder) A**	**Energy of peak (shoulder) A (eV)**
$C_{59}N$	1*s* → LUMO (β spin)	399.96
$C_{59}N(OH)$	1*s* → LUMO	399.97
$(C_{59}N)_2$	1*s* → LUMO	399.94
$C_{59}N$-O-$C_{59}N$	1*s* → LUMO	399.95

Table S2. MO transitions and the corresponding energies for peak A in the oxygen *K*-edge XES of the open [5,6] and closed [6,6] isomers of $C_{60}O$, $C_{59}N(OH)$, and $C_{59}N$-O-$C_{59}N$ molecules.

**Molecule**	**MO transition of peak A**	**Energy of peak A (eV)**
[5,6]$C_{60}O$	HOMO → 1s	515.88
[6,6]$C_{60}O$	HOMO → 1s	515.82
$C_{59}N(OH)$	HOMO → 1s	515.33
$C_{59}N$-O-$C_{59}N$	HOMO → 1s	515.74

Table S3. Assignments of the nitrogen *K*-edge XPS shake-up satellites of the $C_{59}N$, $C_{59}N(OH)$, $(C_{59}N)_2$, and $C_{59}N$-O-$C_{59}N$ molecules.

**Molecule**	**A**	**B**
$C_{59}N$	HOMO→ LUMO+ 2(α)	HOMO– 5 → LUMO+ 2(α)
$C_{59}N(OH)$	HOMO→ LUMO+ 2	HOMO→ LUMO+ 6
		HOMO–5→ LUMO+ 2
		HOMO–4→ LUMO+ 4
$(C_{59}N)_2$	HOMO→ LUMO+ 9	HOMO– 6 → LUMO+ 8
	HOMO –5→ LUMO+ 2	HOMO– 18 → LUMO+ 2
$C_{59}N$-O-$C_{59}N$	HOMO→ LUMO+ 6	HOMO–6→ LUMO+ 8
	HOMO–9→ LUMO	HOMO–9→ LUMO+ 2
		HOMO–12→ LUMO+ 6

Table S4. Assignments of the oxygen *K*-edge XPS shake-up satellites of the open [5,6] and closed [6,6] isomers of $C_{60}O$, $C_{60}H(OH)$, C_{60}-O-C_{60}, $C_{60}H$-O-$C_{60}H$, $C_{59}N(OH)$, and $C_{59}N$-O-$C_{59}N$ molecules.

Molecule	A	B	C
[5,6]$C_{60}O$	HOMO − 3 → LUMO + 1<br>HOMO − 4 → LUMO	—	HOMO − 9 → LUMO + 1<br>HOMO − 8 → LUMO + 1
[6,6]$C_{60}O$	HOMO − 4 → LUMO	HOMO − 4 → LUMO + 3	HOMO − 8 → LUMO + 2
$C_{60}H(OH)$	HOMO → LUMO	HOMO − 4→ LUMO	HOMO − 5 → LUMO
C_{60}-O-C_{60}	HOMO − 2→ LUMO + 3<br>HOMO → LUMO+ 5<br>HOMO − 8 → LUMO	HOMO − 12 → LUMO	HOMO − 12 → LUMO + 3<br>HOMO − 10 → LUMO + 5
$C_{60}H$-O-$C_{60}H$	HOMO − 4 → LUMO	HOMO −5 → LUMO + 4<br>HOMO → LUMO + 5	HOMO − 12 → LUMO
$C_{59}N(OH)$	HOMO → LUMO	—	HOMO − 5 → LUMO
$C_{59}N$-O-$C_{59}N$	HOMO −2→ LUMO + 1<br>HOMO − 2→ LUMO + 3<br>HOMO → LUMO + 5	—	HOMO − 11 → LUMO + 3<br>HOMO − 10 → LUMO + 5

Table S5. The lowest frequencies of all optimized structures.

Molecule	lowest (1) (cm^{-1})	lowest (2) (cm^{-1})	lowest (3) (cm^{-1})	lowest (4) (cm^{-1})	lowest (5) (cm^{-1})
C_{60}	264.6337	264.6340	264.6345	264.6350	264.6355
[5,6]$C_{60}O$	240.0953	241.8540	264.1200	266.9012	268.6820
[6,6]$C_{60}O$	257.8139	258.6538	258.8951	266.2602	267.5548
$C_{60}H(OH)$	191.7129	242.1027	252.0298	255.4117	262.1784
C_{60}-O-C_{60}	14.6776	15.8952	36.8433	94.9132	112.9805
$C_{60}H$-O-$C_{60}H$	3.0406	12.6583	25.2719	64.5702	84.2552
$C_{59}N$	257.8487	261.8285	262.6928	265.9646	268.9307
$C_{59}N(OH)$	181.7198	229.4904	251.5736	259.2045	260.5026
$(C_{59}N)_2$	3.9451	17.9744	19.1445	75.9561	98.5405
$C_{59}N$-O-$C_{59}N$	4.4088	8.8023	20.9036	66.7224	77.9017

Table S6. The energies of the fullerene C_{60}, aza[60]fullerene $C_{59}N$, their oxides, and hydrates molecules at different spin multiplicities. ΔE are the energy differences between the spin triplet (quartet) state and the spin singlet (doublet) state.

Molecule	singlet (a.u.)	doublet (a.u.)	triplet (a.u.)	quartet (a.u.)	ΔE (eV)
C_{60}	-2286.1731	—	-2286.1127	—	1.6436
[5,6]$C_{60}O$	-2361.3595	—	-2361.2957	—	1.7338
[6,6]$C_{60}O$	-2361.3561	—	-2361.2929	—	1.5783
$C_{60}H(OH)$	-2362.5966	—	-2362.5353	—	1.6505
C_{60}-O-C_{60}	-4647.5566	—	-4647.4891	—	1.8387
C_{60}H-O-C_{60}H	-4648.7582	—	-4648.6925	—	1.7867
$C_{59}N$	—	-2302.7913	—	-2302.7265	1.7635
$C_{59}N(OH)$	-2378.6354	—	-2378.5782	—	1.5558
$(C_{59}N)_2$	-4605.6333	—	-4605.5779	—	1.5088
C_{59}N-O-C_{59}N	-4680.8402	—	-4680.7847	—	1.5120

C. Molecules Coordinate

Table S7. Molecules Coordinate of the open [5,6] and closed [6,6] isomers of $C_{60}O$, $C_{60}H(OH)$, C_{60}-O-C_{60}, C_{60}H-O-C_{6}0H, $C_{59}N(OH)$, and C_{59}N-O-C_{59}N molecules.

Molecule	Atom	x	y	z
[5,6]$C_{60}O$	C	0.338440	1.690738	3.081764
	C	1.506536	2.104967	2.331710
	C	1.386131	2.991251	1.249923
	C	0.102088	3.625611	1.075475
	C	-1.048578	3.028019	1.556771
	C	-0.922801	2.095162	2.640554
	C	0.537081	0.319455	3.512067
	C	1.845100	-0.107699	3.048197
	C	2.431872	0.999020	2.311965
	C	3.182547	0.769431	1.167156
	C	2.126932	2.718732	0.000000
	C	-1.048578	3.028019	-1.556771
	C	-2.189512	2.595151	-0.704174
	C	-2.189512	2.595151	0.704174
	C	-2.799361	1.463057	1.406102
	C	-2.023789	1.165148	2.596567
	C	-1.843772	-0.144344	3.037250
	C	-0.533118	-0.578374	3.497333
	C	2.040450	-1.414972	2.607661
	C	0.931778	-2.353674	2.605529
	C	-0.331496	-1.940865	3.038674
	C	-1.519231	-2.351950	2.307090
	C	-2.453044	-1.239311	2.306551
	C	-3.219089	-0.966885	1.174309
	C	-3.399264	0.405225	0.720641
	C	-3.399264	0.405225	-0.720641
	C	-2.799361	1.463057	-1.406102
	C	1.386131	2.991251	-1.249923
	C	-0.533118	-0.578374	-3.497333
	C	0.537081	0.319455	-3.512067
	C	1.845100	-0.107699	-3.048197
	C	2.040450	-1.414972	-2.607661
	C	0.931778	-2.353674	-2.605529

	C	-1.519231	-2.351950	-2.307090
	C	-2.453044	-1.239311	-2.306551
	C	-1.843772	-0.144344	-3.037250
	C	-2.023789	1.165148	-2.596567
	C	-0.922801	2.095162	-2.640554
	C	0.338440	1.690738	-3.081764
	C	2.431872	0.999020	-2.311965
	C	3.182547	0.769431	-1.167156
	C	3.410801	-0.596500	-0.723443
	C	2.846282	-1.665012	-1.423795
	C	2.243994	-2.769534	-0.697436
	C	1.056855	-3.191213	-1.425782
	C	-0.084218	-3.587897	-0.726873
	C	-1.397828	-3.159205	-1.175562
	C	-3.219089	-0.966885	-1.174309
	C	-3.101086	-1.813420	0.000000
	C	-2.208662	-2.888070	0.000000
	C	-1.397828	-3.159205	1.175562
	C	-0.084218	-3.587897	0.726873
	C	1.056855	-3.191213	1.425782
	C	2.243994	-2.769534	0.697436
	C	2.846282	-1.665012	1.423795
	C	3.410801	-0.596500	0.723443
	C	1.506536	2.104967	-2.331710
	C	3.016505	1.624736	0.000000
	C	-0.331496	-1.940865	-3.038674
	C	0.102088	3.625611	-1.075475
	O	-0.017544	4.491316	0.000000
[6,6]$C_{60}O$	C	-2.299383	0.737840	2.501362
	C	-2.299383	-0.737840	2.501362
	C	-1.195160	-1.452753	2.941543
	C	0.000000	-0.768956	3.516743
	C	0.000000	0.768956	3.516743
	C	-1.195160	1.452753	2.941543
	C	-3.017554	1.176500	1.321292
	C	-3.462188	0.000000	0.593610
	C	-3.017554	-1.176500	1.321292
	C	-2.594543	-2.308018	0.615257
	C	-0.727853	-2.585895	2.183636
	C	2.299383	-0.737840	2.501362
	C	2.299383	0.737840	2.501362
	C	1.195160	1.452753	2.941543
	C	0.727853	2.585895	2.183636
	C	-0.727853	2.585895	2.183636
	C	-1.423774	3.030322	1.056329
	C	-2.594543	2.308018	0.615257
	C	-3.471101	0.000000	-0.801034
	C	-3.027197	1.176309	-1.532041
	C	-2.599344	2.308184	-0.837733
	C	-1.425312	3.036188	-1.291663
	C	-0.696999	3.483982	-0.118061
	C	0.696999	3.483982	-0.118061
	C	1.423774	3.030322	1.056329
	C	2.594543	2.308018	0.615257
	C	3.017554	1.176500	1.321292
	C	0.727853	-2.585895	2.183636
	C	3.027197	-1.176309	-1.532041

	C	2.599344	-2.308184	-0.837733
	C	1.425312	-3.036188	-1.291663
	C	0.727030	-2.602801	-2.420184
	C	1.175662	-1.425226	-3.142456
	C	2.304006	0.727479	-2.707408
	C	3.027197	1.176309	-1.532041
	C	3.471101	0.000000	-0.801034
	C	3.462188	0.000000	0.593610
	C	3.017554	-1.176500	1.321292
	C	2.594543	-2.308018	0.615257
	C	0.696999	-3.483982	-0.118061
	C	-0.696999	-3.483982	-0.118061
	C	-1.425312	-3.036188	-1.291663
	C	-0.727030	-2.602801	-2.420184
	C	-1.175662	-1.425226	-3.142456
	C	0.000000	-0.697560	-3.590530
	C	0.000000	0.697560	-3.590530
	C	1.175662	1.425226	-3.142456
	C	2.599344	2.308184	-0.837733
	C	1.425312	3.036188	-1.291663
	C	0.727030	2.602801	-2.420184
	C	-0.727030	2.602801	-2.420184
	C	-1.175662	1.425226	-3.142456
	C	-2.304006	0.727479	-2.707408
	C	-2.304006	-0.727479	-2.707408
	C	-3.027197	-1.176309	-1.532041
	C	-2.599344	-2.308184	-0.837733
	C	1.423774	-3.030322	1.056329
	C	-1.423774	-3.030322	1.056329
	C	2.304006	-0.727479	-2.707408
	C	1.195160	-1.452753	2.941543
	O	0.000000	0.000000	4.712202
$C_{60}H(OH)$	C	1.959187	-2.868579	1.175359
	C	2.984529	-1.943128	0.726945
	C	3.204631	-0.753716	1.424780
	C	2.406040	-0.441584	2.599072
	C	1.418756	-1.332246	3.026237
	C	1.193073	-2.568597	2.303818
	C	1.326125	-3.442389	0.000000
	C	1.959187	-2.868579	-1.175359
	C	2.984529	-1.943128	-0.726945
	C	3.204631	-0.753716	-1.424780
	C	3.430167	0.482498	0.696766
	C	2.140496	0.985202	2.593355
	C	0.894269	1.468369	3.006578
	C	-0.128878	0.539904	3.452197
	C	0.127274	-0.830874	3.469488
	C	-0.895731	-1.765427	3.027818
	C	-0.238126	-2.835661	2.303833
	C	-0.844123	-3.392180	1.175445
	C	-0.046150	-3.697659	0.000000
	C	1.193073	-2.568597	-2.303818
	C	-0.238126	-2.835661	-2.303833
	C	-0.844123	-3.392180	-1.175445
	C	-2.134382	-2.899270	-0.727081
	C	-2.134382	-2.899270	0.727081
	C	-2.768956	-1.869844	1.424956

	C	-2.137243	-1.290598	2.599617
	C	-2.407342	0.135760	2.595620
	C	-1.419042	1.037132	3.007086
	C	2.767678	1.554062	1.424490
	C	0.857445	3.105318	-1.181648
	C	0.249499	2.541764	-2.281616
	C	-1.206363	2.272558	-2.286821
	C	-2.954376	1.627762	-0.725941
	C	-2.954376	1.627762	0.725941
	C	-1.966939	2.569648	1.177533
	C	-1.206363	2.272558	2.286821
	C	0.249499	2.541764	2.281616
	C	0.857445	3.105318	1.181648
	C	2.115444	2.576628	-0.727616
	C	2.767678	1.554062	-1.424490
	C	2.140496	0.985202	-2.593355
	C	0.894269	1.468369	-3.006578
	C	-0.128878	0.539904	-3.452197
	C	-1.419042	1.037132	-3.007086
	C	-2.407342	0.135760	-2.595620
	C	-3.195513	0.439254	-1.426216
	C	-3.195513	0.439254	1.426216
	C	-3.425601	-0.797615	0.697184
	C	-3.425601	-0.797615	-0.697184
	C	-2.768956	-1.869844	-1.424956
	C	-2.137243	-1.290598	-2.599617
	C	-0.895731	-1.765427	-3.027818
	C	0.127274	-0.830874	-3.469488
	C	1.418756	-1.332246	-3.026237
	C	2.406040	-0.441584	-2.599072
	C	2.115444	2.576628	0.727616
	C	3.430167	0.482498	-0.696766
	C	-1.966939	2.569648	-1.177533
	C	0.087731	3.726303	0.000000
	C	-1.461373	3.404780	0.000000
	O	0.138785	5.159327	0.000000
	H	1.070339	5.422345	0.000000
	H	-1.929081	4.398361	0.000000
C_{60}-O-C_{60}	C	-3.027339	5.783460	-1.516255
	C	-3.470201	5.360137	-0.197051
	C	-3.452647	4.007161	0.141202
	C	-3.003777	3.019341	-0.824276
	C	-2.594053	3.426578	-2.098015
	C	-2.599787	4.835119	-2.448054
	C	-2.303993	7.030374	-1.364233
	C	-2.304003	7.382959	0.048395
	C	-3.027535	6.353574	0.768413
	C	-2.599603	5.954122	2.036732
	C	-3.009084	3.589064	1.458438
	C	-2.280394	1.987099	-0.115956
	C	-0.725416	1.841425	-1.974434
	C	-1.424434	2.821969	-2.687411
	C	-0.696801	3.850445	-3.413021
	C	-1.425083	5.098418	-3.263426
	C	-0.727140	6.298469	-3.114667
	C	-1.175442	7.283892	-2.146938
	C	-1.175429	7.974346	0.620116

C	0.000000	8.231743	-0.194411
C	0.000000	7.893824	-1.548735
C	1.175442	7.283892	-2.146938
C	0.727140	6.298469	-3.114667
C	1.425083	5.098418	-3.263426
C	0.696801	3.850445	-3.413021
C	1.424434	2.821969	-2.687411
C	0.725416	1.841425	-1.974434
C	-2.283599	2.345241	1.318651
C	2.283599	2.345241	1.318651
C	1.180306	2.091820	2.099338
C	0.726826	3.089638	3.027405
C	1.424583	4.290123	3.195816
C	3.452647	4.007161	0.141202
C	3.003777	3.019341	-0.824276
C	2.280394	1.987099	-0.115956
C	1.175660	1.392817	-0.684952
C	-0.726826	3.089638	3.027405
C	-1.424583	4.290123	3.195816
C	-0.696936	5.539323	3.352192
C	0.696936	5.539323	3.352192
C	1.424770	6.569776	2.632702
C	2.599603	5.954122	2.036732
C	3.027535	6.353574	0.768413
C	3.470201	5.360137	-0.197051
C	2.594053	3.426578	-2.098015
C	2.599787	4.835119	-2.448054
C	3.027339	5.783460	-1.516255
C	2.303993	7.030374	-1.364233
C	2.304003	7.382959	0.048395
C	1.175429	7.974346	0.620116
C	0.727166	7.559653	1.937687
C	-0.727166	7.559653	1.937687
C	-1.424770	6.569776	2.632702
C	-1.180306	2.091820	2.099338
C	-2.594832	4.546805	2.389990
C	3.009084	3.589064	1.458438
C	2.594832	4.546805	2.389990
C	0.000000	0.808081	0.116535
C	0.000000	-0.808081	0.116535
C	1.175660	-1.392817	-0.684952
C	-1.175660	-1.392817	-0.684952
C	2.280394	-1.987099	-0.115956
C	0.725416	-1.841425	-1.974434
C	1.180306	-2.091820	2.099338
C	-1.180306	-2.091820	2.099338
C	-0.725416	-1.841425	-1.974434
C	-2.280394	-1.987099	-0.115956
C	2.283599	-2.345241	1.318651
C	3.003777	-3.019341	-0.824276
C	1.424434	-2.821969	-2.687411
C	0.726826	-3.089638	3.027405
C	-0.726826	-3.089638	3.027405
C	-2.283599	-2.345241	1.318651
C	-1.424434	-2.821969	-2.687411
C	-3.003777	-3.019341	-0.824276
C	3.009084	-3.589064	1.458438

	C	3.452647	-4.007161	0.141202
	C	2.594053	-3.426578	-2.098015
	C	0.696801	-3.850445	-3.413021
	C	1.424583	-4.290123	3.195816
	C	-1.424583	-4.290123	3.195816
	C	-3.009084	-3.589064	1.458438
	C	-2.594053	-3.426578	-2.098015
	C	-0.696801	-3.850445	-3.413021
	C	-3.452647	-4.007161	0.141202
	C	2.594832	-4.546805	2.389990
	C	3.470201	-5.360137	-0.197051
	C	2.599787	-4.835119	-2.448054
	C	1.425083	-5.098418	-3.263426
	C	0.696936	-5.539323	3.352192
	C	-2.594832	-4.546805	2.389990
	C	-0.696936	-5.539323	3.352192
	C	-2.599787	-4.835119	-2.448054
	C	-1.425083	-5.098418	-3.263426
	C	-3.470201	-5.360137	-0.197051
	C	2.599603	-5.954122	2.036732
	C	3.027535	-6.353574	0.768413
	C	3.027339	-5.783460	-1.516255
	C	0.727140	-6.298469	-3.114667
	C	1.424770	-6.569776	2.632702
	C	-2.599603	-5.954122	2.036732
	C	-1.424770	-6.569776	2.632702
	C	-3.027339	-5.783460	-1.516255
	C	-0.727140	-6.298469	-3.114667
	C	-3.027535	-6.353574	0.768413
	C	2.304003	-7.382959	0.048395
	C	2.303993	-7.030374	-1.364233
	C	1.175442	-7.283892	-2.146938
	C	0.727166	-7.559653	1.937687
	C	-0.727166	-7.559653	1.937687
	C	-2.303993	-7.030374	-1.364233
	C	-1.175442	-7.283892	-2.146938
	C	-2.304003	-7.382959	0.048395
	C	1.175429	-7.974346	0.620116
	C	0.000000	-7.893824	-1.548735
	C	-1.175429	-7.974346	0.620116
	C	0.000000	-8.231743	-0.194411
	C	0.000000	1.210811	1.657056
	C	0.000000	-1.210811	1.657056
	C	-1.175660	1.392817	-0.684952
	O	0.000000	0.000000	2.419174
$C_{60}H$-O-$C_{60}H$	C	1.175557	-6.638021	-2.910960
	C	0.727085	-5.382844	-3.487466
	C	1.424959	-4.203501	-3.219000
	C	2.599094	-4.232198	-2.361661
	C	3.026350	-5.440206	-1.806892
	C	2.304251	-6.665347	-2.088695
	C	0.000000	-7.415091	-2.556139
	C	-1.175557	-6.638021	-2.910960
	C	-0.727085	-5.382844	-3.487466
	C	-1.424959	-4.203501	-3.219000
	C	0.696628	-2.979543	-2.936838
	C	2.591060	-3.027450	-1.552432

C	3.002086	-3.073574	-0.216978
C	3.450222	-4.330414	0.354431
C	3.469273	-5.489004	-0.422458
C	3.028063	-6.751929	0.147724
C	2.303968	-7.475652	-0.879397
C	1.175483	-8.226181	-0.542667
C	0.000000	-8.191546	-1.396288
C	-2.304251	-6.665347	-2.088695
C	-2.303968	-7.475652	-0.879397
C	-1.175483	-8.226181	-0.542667
C	-0.727103	-8.283050	0.837309
C	0.727103	-8.283050	0.837309
C	1.425377	-7.588975	1.827096
C	2.599983	-6.806704	1.475620
C	2.595971	-5.602968	2.287024
C	3.007310	-4.383928	1.735844
C	1.421635	-2.258829	-1.903088
C	-1.184188	-1.566357	0.464068
C	-2.277855	-2.338468	0.799920
C	-2.287350	-3.163703	2.028575
C	-0.725653	-4.451476	3.377742
C	0.725653	-4.451476	3.377742
C	1.176494	-3.197289	2.840374
C	2.287350	-3.163703	2.028575
C	2.277855	-2.338468	0.799920
C	1.184188	-1.566357	0.464068
C	-0.725797	-1.579184	-0.900071
C	-1.421635	-2.258829	-1.903088
C	-2.591060	-3.027450	-1.552432
C	-3.002086	-3.073574	-0.216978
C	-3.450222	-4.330414	0.354431
C	-3.007310	-4.383928	1.735844
C	-2.595971	-5.602968	2.287024
C	-1.426534	-5.637591	3.131397
C	1.426534	-5.637591	3.131397
C	0.697434	-6.864577	2.854463
C	-0.697434	-6.864577	2.854463
C	-1.425377	-7.588975	1.827096
C	-2.599983	-6.806704	1.475620
C	-3.028063	-6.751929	0.147724
C	-3.469273	-5.489004	-0.422458
C	-3.026350	-5.440206	-1.806892
C	-2.599094	-4.232198	-2.361661
C	-0.696628	-2.979543	-2.936838
C	-1.176494	-3.197289	2.840374
C	1.184188	1.566357	0.464068
C	-1.184188	1.566357	0.464068
C	2.277855	2.338468	0.799920
C	0.725797	1.579184	-0.900071
C	1.176494	3.197289	2.840374
C	-1.176494	3.197289	2.840374
C	-2.277855	2.338468	0.799920
C	2.287350	3.163703	2.028575
C	3.002086	3.073574	-0.216978
C	1.421635	2.258829	-1.903088
C	0.725653	4.451476	3.377742
C	-0.725653	4.451476	3.377742

	C	-2.287350	3.163703	2.028575
	C	-1.421635	2.258829	-1.903088
	C	-3.002086	3.073574	-0.216978
	C	3.007310	4.383928	1.735844
	C	3.450222	4.330414	0.354431
	C	2.591060	3.027450	-1.552432
	C	0.696628	2.979543	-2.936838
	C	1.426534	5.637591	3.131397
	C	-1.426534	5.637591	3.131397
	C	-3.007310	4.383928	1.735844
	C	-2.591060	3.027450	-1.552432
	C	-0.696628	2.979543	-2.936838
	C	-3.450222	4.330414	0.354431
	C	2.595971	5.602968	2.287024
	C	3.469273	5.489004	-0.422458
	C	2.599094	4.232198	-2.361661
	C	1.424959	4.203501	-3.219000
	C	0.697434	6.864577	2.854463
	C	-2.595971	5.602968	2.287024
	C	-0.697434	6.864577	2.854463
	C	-2.599094	4.232198	-2.361661
	C	-1.424959	4.203501	-3.219000
	C	-3.469273	5.489004	-0.422458
	C	2.599983	6.806704	1.475620
	C	3.028063	6.751929	0.147724
	C	3.026350	5.440206	-1.806892
	C	0.727085	5.382844	-3.487466
	C	1.425377	7.588975	1.827096
	C	-2.599983	6.806704	1.475620
	C	-1.425377	7.588975	1.827096
	C	-3.026350	5.440206	-1.806892
	C	-0.727085	5.382844	-3.487466
	C	-3.028063	6.751929	0.147724
	C	2.303968	7.475652	-0.879397
	C	2.304251	6.665347	-2.088695
	C	1.175557	6.638021	-2.910960
	C	0.727103	8.283050	0.837309
	C	-0.727103	8.283050	0.837309
	C	-2.304251	6.665347	-2.088695
	C	-1.175557	6.638021	-2.910960
	C	-2.303968	7.475652	-0.879397
	C	1.175483	8.226181	-0.542667
	C	0.000000	7.415091	-2.556139
	C	-1.175483	8.226181	-0.542667
	C	0.000000	8.191546	-1.396288
	C	-0.725797	1.579184	-0.900071
	C	0.725797	-1.579184	-0.900071
	C	0.000000	1.296911	1.416511
	C	0.000000	-1.296911	1.416511
	C	0.000000	-2.235194	2.706054
	C	0.000000	2.235194	2.706054
	O	0.000000	0.000000	2.061802
	H	0.000000	-1.518373	3.536137
	H	0.000000	1.518373	3.536137
$C_{59}N(OH)$	C	1.950493	-2.861800	1.175510
	C	2.978653	-1.939400	0.726807
	C	3.202400	-0.750728	1.424539

C	2.406560	-0.437732	2.600656
C	1.414477	-1.323550	3.026408
C	1.185865	-2.560365	2.304715
C	1.317582	-3.435377	0.000000
C	1.950493	-2.861800	-1.175510
C	2.978653	-1.939400	-0.726807
C	3.202400	-0.750728	-1.424539
C	3.429934	0.484919	0.696365
C	2.145009	0.989485	2.597471
C	0.904003	1.473858	3.021294
C	-0.125606	0.554696	3.466098
C	0.124168	-0.818265	3.472870
C	-0.901067	-1.750409	3.032705
C	-0.245787	-2.820940	2.304117
C	-0.852662	-3.374851	1.175685
C	-0.056045	-3.682622	0.000000
C	1.185865	-2.560365	-2.304715
C	-0.245787	-2.820940	-2.304117
C	-0.852662	-3.374851	-1.175685
C	-2.140902	-2.875147	-0.727487
C	-2.140902	-2.875147	0.727487
C	-2.772748	-1.846639	1.427216
C	-2.138533	-1.268606	2.603002
C	-2.408712	0.158218	2.605925
C	-1.416597	1.061986	3.017091
C	2.768458	1.558582	1.425441
C	0.859717	3.107802	-1.186114
C	0.261071	2.545525	-2.291563
C	-1.182228	2.295654	-2.302393
C	-2.937743	1.636386	-0.717066
C	-2.937743	1.636386	0.717066
C	-1.894269	2.530191	1.136309
C	-1.182228	2.295654	2.302393
C	0.261071	2.545525	2.291563
C	0.859717	3.107802	1.186114
C	2.115132	2.578600	-0.726386
C	2.768458	1.558582	-1.425441
C	2.145009	0.989485	-2.597471
C	0.904003	1.473858	-3.021294
C	-0.125606	0.554696	-3.466098
C	-1.416597	1.061986	-3.017091
C	-2.408712	0.158218	-2.605925
C	-3.188909	0.459328	-1.432649
C	-3.188909	0.459328	1.432649
C	-3.427159	-0.772774	0.698452
C	-3.427159	-0.772774	-0.698452
C	-2.772748	-1.846639	-1.427216
C	-2.138533	-1.268606	-2.603002
C	-0.901067	-1.750409	-3.032705
C	0.124168	-0.818265	-3.472870
C	1.414477	-1.323550	-3.026408
C	2.406560	-0.437732	-2.600656
C	2.115132	2.578600	0.726386
C	3.429934	0.484919	-0.696365
C	-1.894269	2.530191	-1.136309
C	0.052879	3.679838	0.000000
N	-1.331155	3.146329	0.000000

	O	-0.090428	5.090277	0.000000
	H	0.798822	5.474122	0.000000
$C_{59}N$-O-$C_{59}N$	C	-1.175591	6.880127	-2.732438
	C	-0.726916	5.680890	-3.417827
	C	-1.424775	4.482457	-3.255395
	C	-2.600919	4.436087	-2.401443
	C	-3.026792	5.589250	-1.739096
	C	-2.305003	6.835122	-1.911807
	C	0.000000	7.623617	-2.312345
	C	1.175591	6.880127	-2.732438
	C	0.726916	5.680890	-3.417827
	C	1.424775	4.482457	-3.255395
	C	-0.696311	3.238491	-3.081105
	C	-2.596447	3.164490	-1.702660
	C	-3.018696	3.094745	-0.372476
	C	-3.465685	4.291057	0.314281
	C	-3.472988	5.515681	-0.355341
	C	-3.032928	6.723288	0.323991
	C	-2.304349	7.533240	-0.635194
	C	-1.175717	8.249838	-0.233680
	C	0.000000	8.290994	-1.086529
	C	2.305003	6.835122	-1.911807
	C	2.304349	7.533240	-0.635194
	C	1.175717	8.249838	-0.233680
	C	0.727497	8.181241	1.146390
	C	-0.727497	8.181241	1.146390
	C	-1.427483	7.404438	2.070026
	C	-2.603555	6.656131	1.650279
	C	-2.606556	5.386835	2.355787
	C	-3.018782	4.216456	1.699465
	C	-1.423704	2.428957	-2.114333
	C	1.188120	1.547211	0.183752
	C	2.290101	2.277972	0.575590
	C	2.304365	2.970736	1.865295
	C	0.716944	4.151714	3.322140
	C	-0.716944	4.151714	3.322140
	C	-1.136552	2.976583	2.610939
	C	-2.304365	2.970736	1.865295
	C	-2.290101	2.277972	0.575590
	C	-1.188120	1.547211	0.183752
	C	0.725105	1.667664	-1.173170
	C	1.423704	2.428957	-2.114333
	C	2.596447	3.164490	-1.702660
	C	3.018696	3.094745	-0.372476
	C	3.465685	4.291057	0.314281
	C	3.018782	4.216456	1.699465
	C	2.606556	5.386835	2.355787
	C	1.432952	5.347523	3.191140
	C	-1.432952	5.347523	3.191140
	C	-0.698582	6.591621	3.029281
	C	0.698582	6.591621	3.029281
	C	1.427483	7.404438	2.070026
	C	2.603555	6.656131	1.650279
	C	3.032928	6.723288	0.323991
	C	3.472988	5.515681	-0.355341
	C	3.026792	5.589250	-1.739096
	C	2.600919	4.436087	-2.401443

C	0.696311	3.238491	-3.081105
C	1.136552	2.976583	2.610939
C	-1.188120	-1.547211	0.183752
C	1.188120	-1.547211	0.183752
C	-2.290101	-2.277972	0.575590
C	-0.725105	-1.667664	-1.173170
C	-1.136552	-2.976583	2.610939
C	1.136552	-2.976583	2.610939
C	2.290101	-2.277972	0.575590
C	-2.304365	-2.970736	1.865295
C	-3.018696	-3.094745	-0.372476
C	-1.423704	-2.428957	-2.114333
C	-0.716944	-4.151714	3.322140
C	0.716944	-4.151714	3.322140
C	2.304365	-2.970736	1.865295
C	1.423704	-2.428957	-2.114333
C	3.018696	-3.094745	-0.372476
C	-3.018782	-4.216456	1.699465
C	-3.465685	-4.291057	0.314281
C	-2.596447	-3.164490	-1.702660
C	-0.696311	-3.238491	-3.081105
C	-1.432952	-5.347523	3.191140
C	1.432952	-5.347523	3.191140
C	3.018782	-4.216456	1.699465
C	2.596447	-3.164490	-1.702660
C	0.696311	-3.238491	-3.081105
C	3.465685	-4.291057	0.314281
C	-2.606556	-5.386835	2.355787
C	-3.472988	-5.515681	-0.355341
C	-2.600919	-4.436087	-2.401443
C	-1.424775	-4.482457	-3.255395
C	-0.698582	-6.591621	3.029281
C	2.606556	-5.386835	2.355787
C	0.698582	-6.591621	3.029281
C	2.600919	-4.436087	-2.401443
C	1.424775	-4.482457	-3.255395
C	3.472988	-5.515681	-0.355341
C	-2.603555	-6.656131	1.650279
C	-3.032928	-6.723288	0.323991
C	-3.026792	-5.589250	-1.739096
C	-0.726916	-5.680890	-3.417827
C	-1.427483	-7.404438	2.070026
C	2.603555	-6.656131	1.650279
C	1.427483	-7.404438	2.070026
C	3.026792	-5.589250	-1.739096
C	0.726916	-5.680890	-3.417827
C	3.032928	-6.723288	0.323991
C	-2.304349	-7.533240	-0.635194
C	-2.305003	-6.835122	-1.911807
C	-1.175591	-6.880127	-2.732438
C	-0.727497	-8.181241	1.146390
C	0.727497	-8.181241	1.146390
C	2.305003	-6.835122	-1.911807
C	1.175591	-6.880127	-2.732438
C	2.304349	-7.533240	-0.635194
C	-1.175717	-8.249838	-0.233680
C	0.000000	-7.623617	-2.312345

C	1.175717	-8.249838	-0.233680
C	0.000000	-8.290994	-1.086529
C	0.725105	-1.667664	-1.173170
C	-0.725105	1.667664	-1.173170
C	0.000000	-1.262251	1.129499
C	0.000000	1.262251	1.129499
N	0.000000	2.221743	2.262717
N	0.000000	-2.221743	2.262717
O	0.000000	0.000000	1.807137
